# Metastasis to the appendix from adenocarcinoma of the ascending colon: A case report

**DOI:** 10.1097/MD.0000000000006357

**Published:** 2017-03-24

**Authors:** Yingjie Li, Mingshan Li, Xiaoxia Li, Haiquan Sang

**Affiliations:** Department of General Surgery, The Fourth Affiliated Hospital of China Medical University, Shenyang, Liaoning, People's Republic of China.

**Keywords:** colon cancer, metastasis to appendix, right hemicolectomy

## Abstract

**Rationale:**

Metastasis of cancer cells involves shedding from the primary tumor through various means to distant tissues and organs with continued growth and formation of new metastatic tumors of the same cancer type as the original tumor. The common sites for colon cancer metastases include the pelvis, retroperitoneal lymph nodes, liver, and lungs; Colon cancer metastases to the appendix are rare, as reported in this case.

**Patient concerns and diagnoses:**

A 45-year-old man was admitted to our department with a 24-hour history of abdominal distension and incomplete obstruction. Colonoscopy showed an elevated lesion in the ascending colon and the pathologic diagnosis was adenocarcinoma.

**Interventions and outcomes:**

This patient underwent a radical right hemi-colectomy. The post-operative pathologic examination revealed metastatic adenocarcinoma in all layers of the appendix, especially the muscularis mucosae. The diagnosis was adenocarcinoma of the ascending colon (pT4bN2bM0 stage IIIC) with metastatic adenocarcinoma of the appendix.

**Lessons:**

An absent right colic artery with lymph node fusion might increase the risk of appendiceal cancer metastasis.

## 1 Introduction

Metastasis of cancer cells involves shedding from the primary tumor through various means to distant tissues and organs with continued growth and formation of new metastatic tumors of the same cancer type as the original tumor. The spread of colon cancer is generally divided into the following four types: direct invasion, seeding, lymphatic metastasis, and hematogenous metastasis.^[1]^ The common sites for colon cancer metastases include the pelvis, retroperitoneal lymph nodes, liver, and lungs; metastases to the appendix are very rare.

## 2 Case presentation

A written informed consent was obtained, and institutional Ethics Committee of The Fourth Affiliated Hospital of China Medical University approved this case report.

**Figure 1:**
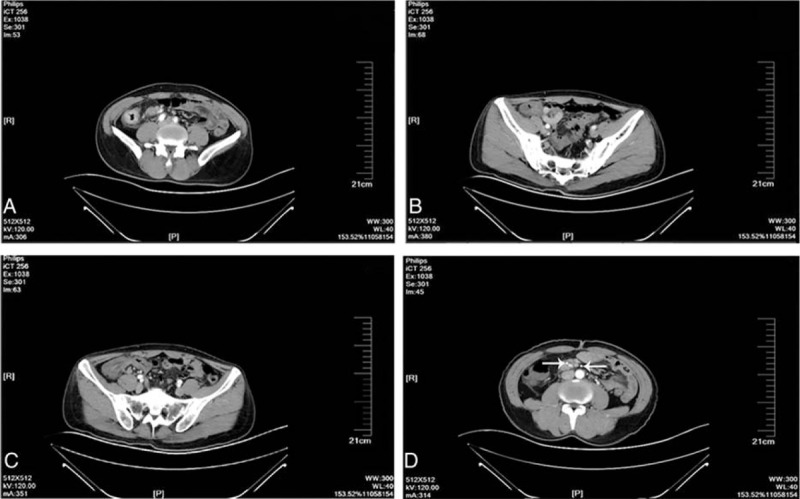
Enhanced abdominal computed tomography images. (A) Ascending bowel wall thickening; (B) cecum and ileum wall thickening; (C) normal mucosa between the colon and appendix; and (D) ileocolic artery and middle colic artery, and right colic artery was absent.

**Figure 2:**
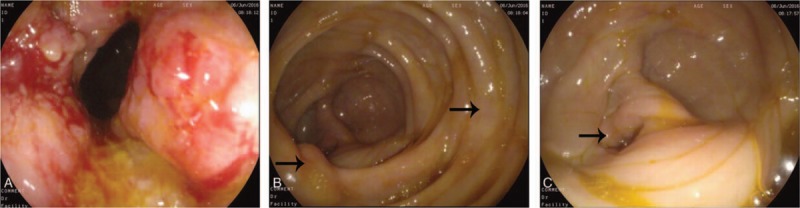
Colonoscopy images. (A) Ascending colon lesions; (B) ileocecal valve and the mucosa between the colon cancer and appendix was normal; and (C) appendiceal orifice.

**Figure 3:**
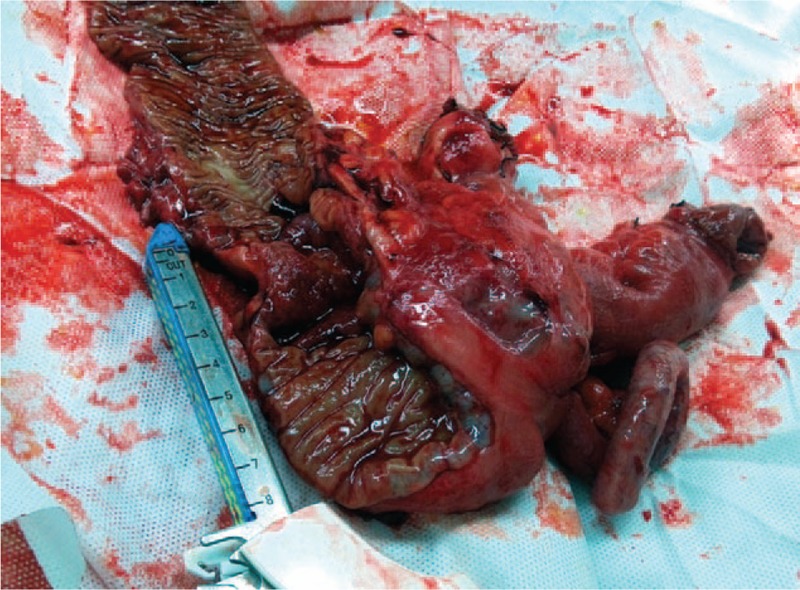
Surgical excision specimens. The intestinal membrane was smooth between the tumor and the appendix.

**Figure 4:**
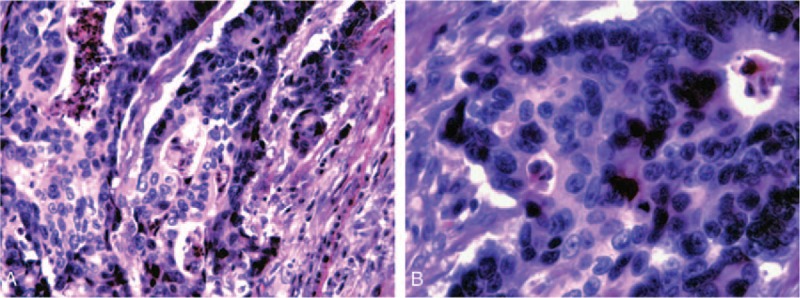
Postoperative pathological images of colon. (A) Pathology of colon cancer (×200); and (B) pathology of colon cancer (×400).

**Figure 5:**
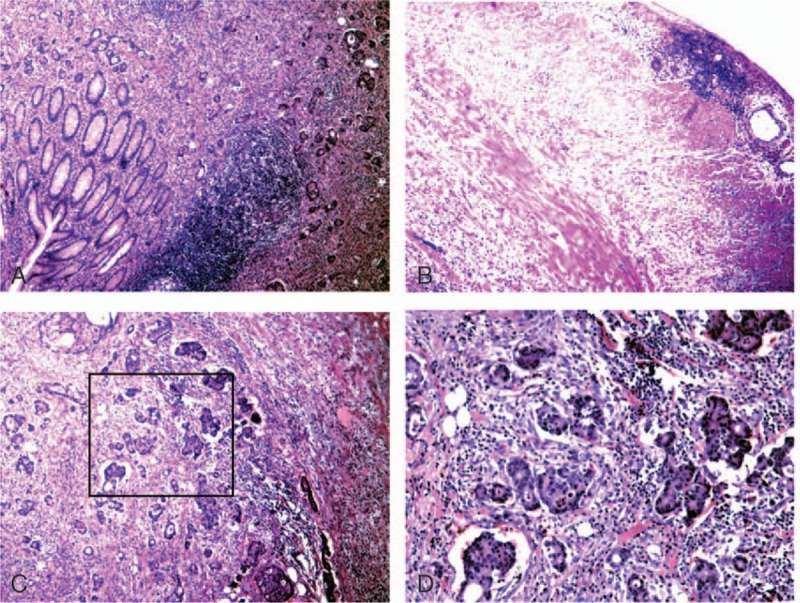
Postoperative pathological images of appendix. (A) The mucosa of the appendix was normal (×40); (B) the serosa of the appendix was normal (×40); (C) pathology of the appendix, metastatic adenocarcinoma was mainly in the muscularis mucosae (×40); (D) pathology of the appendix, local magnification of C, metastatic adenocarcinoma was concentrated in the vascular system (×100).

A 45-year-old man was admitted to our department with a 24-hour history of abdominal distension and incomplete obstruction. On physical examination, a bulge was noted in the right lower quadrant, a 10 × 15 cm hard, immobile mass was palpated, and lower abdominal pain with guarding without rebound tenderness was demonstrated. Bowel sounds were active. There was no family history of cancer. The fecal occult blood test was positive; carcinoembryonic antigen (CEA) and alpha fetoprotein levels were in the normal range. The intestinal incomplete obstruction was treated, after which an enhanced abdominal computed tomography (CT) scan was done. The CT showed the following: focal wall thickening of the ascending colon; multiple enlarged lymph nodes on the right side of the abdominal fat space and retroperitoneum; and the walls of the proximal ascending colon, cecum, and terminal ileum were thickened (Fig. 1). Colonoscopy showed an elevated lesion in the ascending colon (Fig. 2); the pathologic diagnosis was adenocarcinoma. A radical right hemicolectomy was performed. Surgical exploration revealed the following: the tumor corresponded to node clusters in the mesenteric lymph nodes; partial fusion of the mesenteric lymph nodes was noted in the small mesenteric and mesocolon roots; the right colic artery was absent; the appendix was congested, dark red, and firm; and there were no adhesions involving the surrounding tissues. Opening of the surgical specimens disclosed the following: in the middle of ascending colon, 5 cm distal to the ileocecal valve, there was an ulcerative, annular, invasive lesion 4 cm in length and two-thirds of the annular bowel wall in width; the lumen was narrow with a hard texture; and the tumor invaded through the muscularis propria and into the serosa. The appendix was 12 cm in length and 1.3 cm in diameter. The intestinal membrane was smooth between the tumor and the appendix (Fig. 3). The immunohistochemistry results were as follows: CK20 (+); CEA (+); Syn (–); CgA (–); CD56 (weak +); MHL1 (+); MSH2 (+); MSH6 (+); PMS2 (+); CD34 (vascular +); S-100 (+); and Ki-67 (+>80%). The pathologic findings were as follows: right colon, poorly differentiated adenocarcinoma with tumor infiltration and invasion into the serosa and local penetration with tumor thrombus in the vessel and nerve tissue invasion; cut edge with no tumor tissue; metastatic adenocarcinoma in all layers of the appendix, especially the muscularis mucosae; and 16 of 21 lymph node metastases (201 group, 5/6; 202 group, 1/3; and 203, group 10/12; Figs. 4 and 5). The combined pathologic stage was pT4bN2bM0, stage IIIc.

## 3 Discussion

Appendiceal cancer does not generally cause clinical symptoms or present with symptoms of acute or perforated appendicitis. Most cases of appendiceal tumors are detected at the time of appendectomy and postoperative pathologic findings.^[2,3]^ The biological features and histological characteristics between primary appendiceal adenocarcinoma and metastatic carcinoma of the appendix are different.^[2]^ The primary tumor cell infiltrates from the appendiceal mucosa to the muscularis mucosae and serosa are well known. Metastatic cancer of the appendix is rare; most cases have metastasized by peritoneal seeding. In such cases, microscopy reveals gradual serosa invasion.^[4]^ This patient had no symptoms related to appendix. The postoperative pathologic evaluation demonstrated tumor cells concentrated in the mucosae muscularis of the appendix, and a small number of tumor cells were present in the mucosa and serosa (Fig. 4). Thus, the tumor was considered to have undergone lymphatic or hematogenous metastatic spread, rather than representing a primary appendiceal gland tumor or peritoneal metastases. The right colic artery was absent in this patient (Fig. 1). There may be repeated stimulation of recurrent peri-appendiceal asymptomatic inflammation in patients with colon cancer that affects the blood supply and lymphatic drainage of the ascending colon and appendix, which causes metastasis to the appendix. The tumor in this patient may have spread by intraluminal seeding, but the probability of spread was very low. Metastatic location usually accompanies mucosal damage such as hemorrhoidectomy wound;^[5]^ however, there are no reported cases of intraluminal seeding of appendiceal cancer. In the present case, the distance from the appendix metastases to the primary colon tumor was 5 cm (Figs. 2 and 3). The maximum infiltration distance of mucosal metastases in colon cancer is 2 cm.^[6]^ In our patient, the mucosal tissue between the primary colon cancer and appendix was normal, so there was an unlikely probability of mucosal metastases.

## 4 Conclusions

In the present case, an absent right colic artery with lymph node fusion might increase the risk of appendiceal cancer metastasis. If an absent right colic artery and lymph node fusion is noted intraoperatively, then we should pay attention to the appendix because tumor might present when the appendix is congested, thickened, or firm. This case is very rare. It reminds us pay enough attention to details during operation to avoid tumor metastasis.
